# Non–Muscle Invasive Papillary Urothelial Carcinoma Metastatic to the Mandible

**DOI:** 10.1177/2324709618806332

**Published:** 2018-10-16

**Authors:** Noah Frydenlund, Yousef Zakharia, Rohan Garje, Laila Dahmoush, Michael A. O’Donnell

**Affiliations:** 1 The University of Iowa, Iowa City, IA, USA; 2 University of Iowa Hospitals and Clinics, Iowa City, IA, USA

**Keywords:** urothelial carcinoma, GATA-3, mandibular metastasis, surveillance

## Abstract

Urothelial carcinoma, the most common histologic subtype of bladder cancer in the United States, most frequently presents as non–muscle invasive disease. Initially, therapy involves transurethral endoscopic resection and subsequent intravesical therapies with extended surveillance for high-risk disease. Even with the best treatments, recurrence and progression can occur. However, metastasis of non–muscle invasive bladder cancer to distant sites without evidence of progression or regional metastasis is rare. In this article, we present the case of a patient with high-grade papillary urothelial carcinoma who developed an unusual metastasis to the mandible, confirmed by GATA-3 immunostaining, over 4 years after initial transurethral resection. Prior to the development of metastatic disease, this patient had no evidence of local recurrence during maintenance Bacillus Calmette-Guerin intravesical therapy and concurrent surveillance. Positron emission tomography-computed tomography taken after presentation with mandibular metastasis did not show any evidence of regional metastasis. This case highlights an unusual location for distant metastasis of urothelial carcinoma occurring in a patient without evidence of muscle invasive disease or regional metastasis. We additionally highlight the utility of GATA-3 immunostaining in identifying urothelial carcinoma histologically.

## Introduction

Urothelial carcinoma (UC) is the most common subtype of bladder cancer in the United States, and approximately 75% of newly diagnosed cases of UC present with disease confined to the epithelium or lamina propria, known as non–muscle invasive bladder cancer (NMIBC).^1^ Staging of NMIBC is based on histologic findings after transurethral resection of bladder tumor (TURBT), in which tumors are differentiated into papillary carcinoma confined to the epithelial mucosa (Ta), tumors invading into the lamina propria (T1), and carcinoma in situ (Tis).^2^ In addition to clinical staging, histologic grade is used to identify more aggressive lesions. In 2004, the World Health Organization classification for tumor grade was updated, allowing for NMIBC to be classified as either papillary urothelial neoplasm of low malignant potential, low-grade, or high-grade, with increasing grade showing progressively decreased differentiation and increased aggressiveness. The most recent 2016 World Health Organization classification maintains this system.^3^ Increased stage and higher grade disease are associated with an increased risk of recurrence and progression.^1^

While initial TURBT can be curative for low-risk disease, recurrence and progression remain problematic for patients with high-risk disease. As such, current American Urologic Association (AUA) and Society for Urologic Oncology (SUO) guidelines for high-risk NMIBC recommend a second TURBT for restaging within 6 weeks of initial TURBT, followed by induction with Bacillus Calmette-Guerin (BCG) and subsequent maintenance therapy in responders.^2^ High-risk patients are also recommended to undergo extended surveillance with frequent cystoscopy and cytology. These recommendations are aimed at improving recurrence-free survival and progression-free survival as muscle invasive disease has a grave prognosis.

## Case Report

**Figure 1.**
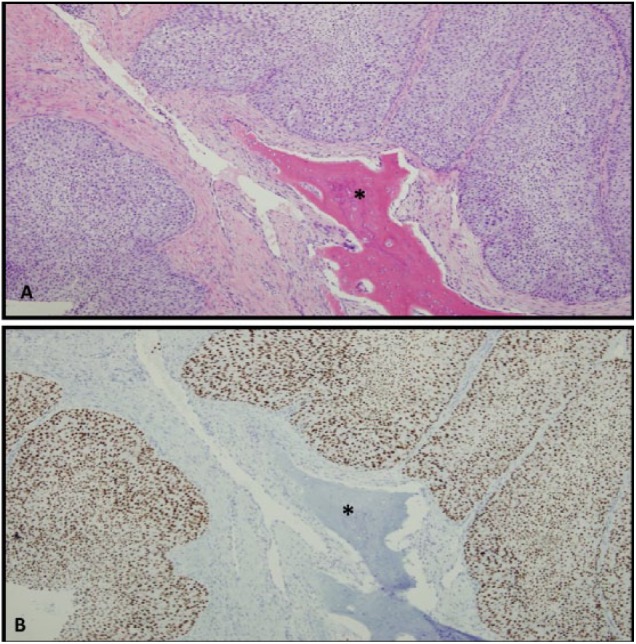
(A) Hematoxylin-eosin stain showing urothelial carcinoma surrounding mandibular bone (asterisk). (B) GATA-3 immunostaining confirming urothelial origin of tumor.

A 56-year-old Caucasian male with a history of non–muscle invasive UC, hypertension, and type 2 diabetes mellitus presented to an oral surgeon with 3 weeks of swelling and pain in his right anterior mandible. Up to that point, his mandibular lesion had been unresponsive to amoxicillin or cephalexin. A cone beam radiograph obtained at the time of presentation showed a radiolucent lesion of the mandible concerning for tumor. An incisional biopsy was performed, and a sample was submitted to our institution for pathologic evaluation. Microscopic examination of hematoxylin and eosin stained sections showed UC, which was confirmed by a positive GATA-3 immunostain, consistent with metastatic UC (Figure 1).

The patient was initially diagnosed with UC 4.5 years prior during evaluation for gross hematuria. Initial cystoscopy showed a 3-cm anterior bladder wall tumor near the bladder neck, a 1- to 2-cm left anterior wall tumor, and a 1-cm right anterior wall tumor. Subsequent TURBT was performed with collected specimens demonstrating noninvasive high-grade papillary UC (TaHG). Muscularis propria was present in all specimens. Given the multiple foci of TaHG lesions, the patient was classified as high-risk. Following TURBT, the patient underwent induction with intravesical BCG and interferon (IFN). Postinduction TURBT was negative for evidence of tumor and both cytology, and fluorescent in situ hybridization (FISH) studies were additionally negative at that time. The patient was scheduled to receive 3 maintenance cycles of intravesical BCG/IFN; however, therapy was discontinued after the second cycle due to development of scrotal swelling and fever. Throughout BCG/IFN maintenance the patient underwent surveillance cystoscopy with cytology and FISH analysis every 3 months for 2 years before spacing surveillance to 6 months. All studies (cystoscopy, cytology, and FISH) performed during surveillance were negative, with no evidence of local recurrence. Additionally, the patient underwent a computed tomography (CT) Urogram at 18 months after his initial TURBT, which was negative for evidence of tumor.

**Figure 2.**
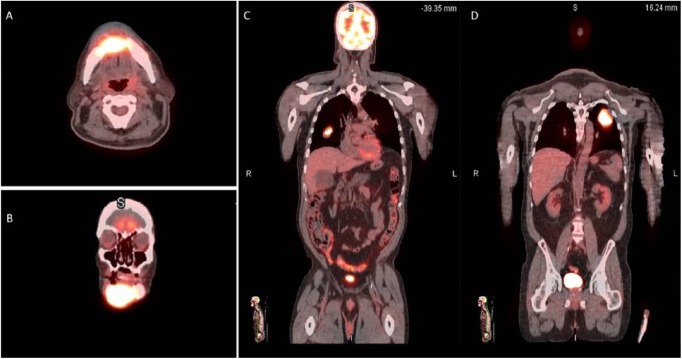
Axial (A) and coronal (B) positron emission tomography-computed tomography (PET-CT) demonstrating a metabolically active lesion in the anterior mandible with body PET-CT (C, D) demonstrating hypermetabolic lesions in the lung concerning for metastasis. Note the absence of hypermetabolic lesions in the pelvis.

After metastatic UC was identified in the mandibular biopsy, the patient underwent a fluorodexoyglucose positron emission tomography (PET)-CT scan that showed a destructive hypermetabolic soft tissue lesion at the mandibular symphysis without evidence of lymphadenopathy in the neck and bilateral hypermetabolic lung masses concerning for metastases (Figure 2). Notably, no hypermetabolic lesions were noted in the abdomen or pelvis. The patient was presented at our institution’s urology and genitourinary oncology tumor board and was subsequently referred to medical oncology and radiation oncology for management. At present, the patient is undergoing palliative radiotherapy to the mandible and is scheduled to begin gemcitabine/cisplatin systemic chemotherapy.

## Discussion

In the case presented here, the presence of a multifocal TaHG tumor is considered high risk under the most recent AUA/SUO risk stratification for NMIBC.^2^ Currently, the AUA/SUO guidelines recommend that high-risk patients with NMIBC undergo induction BCG therapy, followed by BCG maintenance to decrease the risk of both recurrence and progression. While this patient was unable to complete a full course of maintenance BCG, all surveillance cystoscopy, cytology, and FISH studies failed to show evidence of recurrence or progression. The patient had no evidence of residual disease until presenting with biopsy confirmed mandibular metastasis 4.5 years later.

The most common reported sites of metastasis for UC are liver (47%), lung (45%), or bone (32%).^4^ While bone may be a frequent site of metastasis, the bones of the skull are infrequently involved. We report here the 13th known case of UC metastatic to the jaw.^5-15^ Of those cases, we were able to identify only one that mentioned stage (pT3) at the time of tumor discovery.^6^ The majority of jaw metastasis appear to occur in the mandible, with most jaw metastatic disease associated with additional distant metastases (both to bone and soft tissue).^7^ A recent review of 453 cases of metastasis to jawbones suggests that the mandible is the most common site of jaw metastasis regardless of the type of primary tumor, with 74% of cases demonstrating mandibular involvement.^16^ It was also noted that the posterior mandible was more frequently involved than the anterior region, which is hypothesized to be a result of the presence of red marrow posteriorly. In a 2007 study of 390 cases of oral cavity metastasis from a variety of malignancies including UC, the mean time from diagnosis of primary disease to presentation with oral cavity metastasis was 40 months, with oral metastasis associated with a poor prognosis (mean survival of 7 months after presentation).^17^ Although metastasis to the jaw is a rare occurrence, it should be included in the differential and appropriately worked up in patients with a history of malignant diseases who present with lesions of the oral cavity.

Notably, this patient never demonstrated local recurrence or muscle invasive disease during the course of his treatment and surveillance. Additionally, PET-CT following presentation with mandibular metastasis did not show hypermetabolic lesions in the pelvis. At least 2 cases of distant bone metastasis in non–muscle invasive disease have been reported in the literature.^4,18^ In one case, a patient with stage T1HG showed no evidence of lymphovascular invasion or muscle invasive disease at the initial TURBT or the subsequent restaging TURBT.^18^ As the patient’s primary tumor location made complete resection difficult, a partial cystectomy was performed, and lymphadenectomy at that time showed no evidence of regional metastasis. However, 3 months after surgery the patient presented with metastasis to the left iliac wing, vertebrae, and femur. Another recently published case described a patient with T1 disease treated by TURBT and BCG therapy who displayed no signs of recurrence or progression until presenting 3 years later with bony metastasis to the vertebra and femur.^18^ There have been at least 15 other isolated reports of distant NMIBC metastasis to the lungs, liver, ovary, and brain, without evidence of regional metastasis or progression, with at least 3 of these cases reported as being stage Ta.^19^ One interpretation of these findings is that metastasis by hematogenous spread may rarely occur early in the disease course, before UC progresses beyond stage T1. Alternatively, it is possible that areas of muscle invasive tumor may be missed during TURBT.

In this case, the identification of metastatic UC in the jaw biopsy was aided by immunohistochemical staining for the GATA-3 protein. GATA-3 is a zinc-finger transcription factor that binds the DNA consensus sequence G-A-T-A.^20^ Its utility in identifying UC was discovered in 2007 by Higgens et al who utilized complementary DNA microarray studies to identify markers distinguishing UC from prostate carcinoma.^21^ It is now known that GATA-3 has a high sensitivity and specificity for UC, with studies reporting between 76% and 100% expression.^22^ It has additionally been shown that there is a strong concordance between GATA-3 expression in the primary UC lesion and regional metastasis.^23^ However, we were not able to identify any reports regarding the concordance of GATA-3 expression between primary tumors and distant metastasis. The impact, if any, of GATA-3 expression on prognosis is not known, with studies showing mixed results.^24,25^

In summary, we present here the case of patient with TaHG UC, who presented over 4 years after initial TURBT with a distant metastasis to the mandible. The patient had undergone 2/3 cycles of intravesical BCG/IFN and showed no evidence of recurrent disease during surveillance or of regional metastasis on PET-CT. This case underscores the importance of maintaining a high suspicion of metastatic disease when evaluating patients with a history of high-risk UC, even if they had previously shown no evidence of recurrence or progression.
